# Brain Abscess Caused by *Nocardia farcinica* and Diagnosed by Metagenomic Next-Generation Sequencing: A Case Report

**DOI:** 10.3389/fmed.2022.803554

**Published:** 2022-02-16

**Authors:** Jianhua Yang, Shuhua Xie, Junda Li, Han Xia, Xianghong Liu

**Affiliations:** ^1^Department of Neurology, The Affiliated Ganzhou Hospital of Nanchang University, Ganzhou, China; ^2^Department of Scientific Affairs, Hugobiotech Co., Ltd., Beijing, China

**Keywords:** *Nocardia farcinica*, central nervous system, infection, brain abscess, metagenomic next-generation sequencing

## Abstract

**Background:**

Brain abscesses caused by *Nocardia farcinica* are rare and difficult to diagnose. Conventional methods for diagnosing Nocardia species include blood culture, microscopy, and tissue slice, but the performance is not satisfied. We report a case of brain abscess due to *N. farcinica* diagnosed by metagenomic next-generation sequencing (mNGS).

**Case Presentation:**

We report a case of a 58-year-old man with brain abscess caused by *N. farcinica*. The patient had a history of pemphigus and required long-term methylprednisolone administration. No pathogen was detected in blood culture, cerebrospinal fluid (CSF) culture, and fast-acid staining. mNGS identified *N. farcinica* in the CSF. The symptoms and signs of the patient were significantly improved after changing the antibiotics accordingly to sensitive antibiotics.

**Conclusion:**

Metagenomic next-generation sequencing (mNGS) is helpful for early diagnosis and subsequent treatment of *Nocardia*-associated meningitis and encephalitis, avoiding brain surgery. Early and accurate diagnosis and prompt antibiotic treatment reduced its mortality.

## Background

*Nocardia* is a Gram-positive, branching aerobic bacterium and universal in soil, water, and decaying plant matters. The *Nocardia* bacterium is an opportunistic pathogen that infects through the respiratory tract or skin contact. Predisposing factors are immunosuppressive treatment (28%), premedication (26%), hematological neoplasm (13%), transplant recipients (13%), HIV infection (11%), chronic lung disease (9%), diabetes mellitus (8%), and renal disease (8%) (1). Clinical forms of *Nocardia farcinica* infection include pulmonary or pleural infections, brain abscesses and skin or soft tissue infections (2). Brain abscesses caused by *N. farcinica* are rare and account for 2% of all intracranial abscesses (3). However, the mortality rate of nocardial brain abscesses is as high as 30%, which is much higher than the 10% caused by other bacteria (4), even with the development of imaging technologies and new antibiotics.

The diagnosis of *N. farcinica* using conventional methods is difficult, and the misdiagnosis may lead to inappropriate treatments and delay the therapy, resulting in the high mortality of the disease. Routine diagnostic methods are blood, cerebrospinal fluid, and brain aspirate culture, and craniotomy is generally performed to remove a lesion and obtain a specimen. According to the literature, 45.8% of patients underwent both medical and surgical management (5). Currently, metagenomic next-generation sequencing (mNGS) is being performed to identify pathogens in many infectious diseases, with high sensitivity and specificity (6). The introduction of this technique would contribute to early and accurate diagnosis of the disease, avoid surgery, and reduce mortality. Here, we report a case of *N. farcinica* brain abscess diagnosed by mNGS.

## Case Presentation

A 58-year-old male with slurred speech for 11 days, accompanied by headache, fever, dizziness and vomiting for 1 day, was admitted to the Department of Neurology at the Affiliated Ganzhou Hospital of Nanchang University on November 15, 2018. He had a history of tuberculosis in 1978, pemphigus treated with oral methylprednisolone 8 mg per day for 1 year, and fungal pneumonia treated with voriconazole 200 mg twice daily. On examination, his body temperature was 37.1°C. Neurologic examination revealed nuchal rigidity, dysarthria, and impaired coordination (finger to nose test) of the right upper limb.

**Figure 1:**
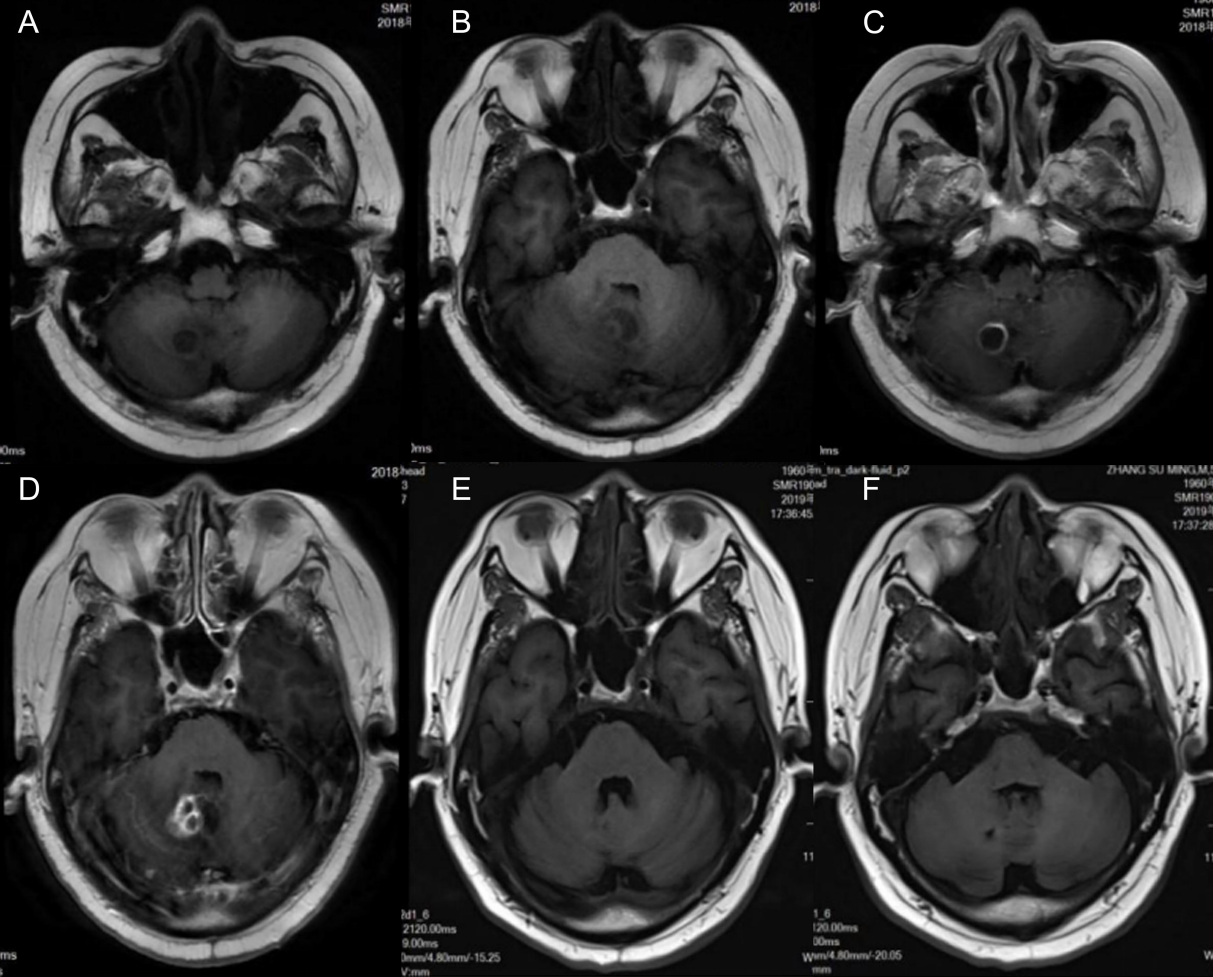
Magnetic resonance imaging (MRI) of the patient shows cerebellar vermis and right cerebellar brain abscess with ring contrast enhancement and peripheral edema. **(A,B)** T1-weighted imaging. **(C,D)** Axial and sagittal T1 contrast enhancement imaging. **(E,F)** T1-weighted imaging 4 months after treatment; the lesion was reduced markedly.

**Figure 2:**
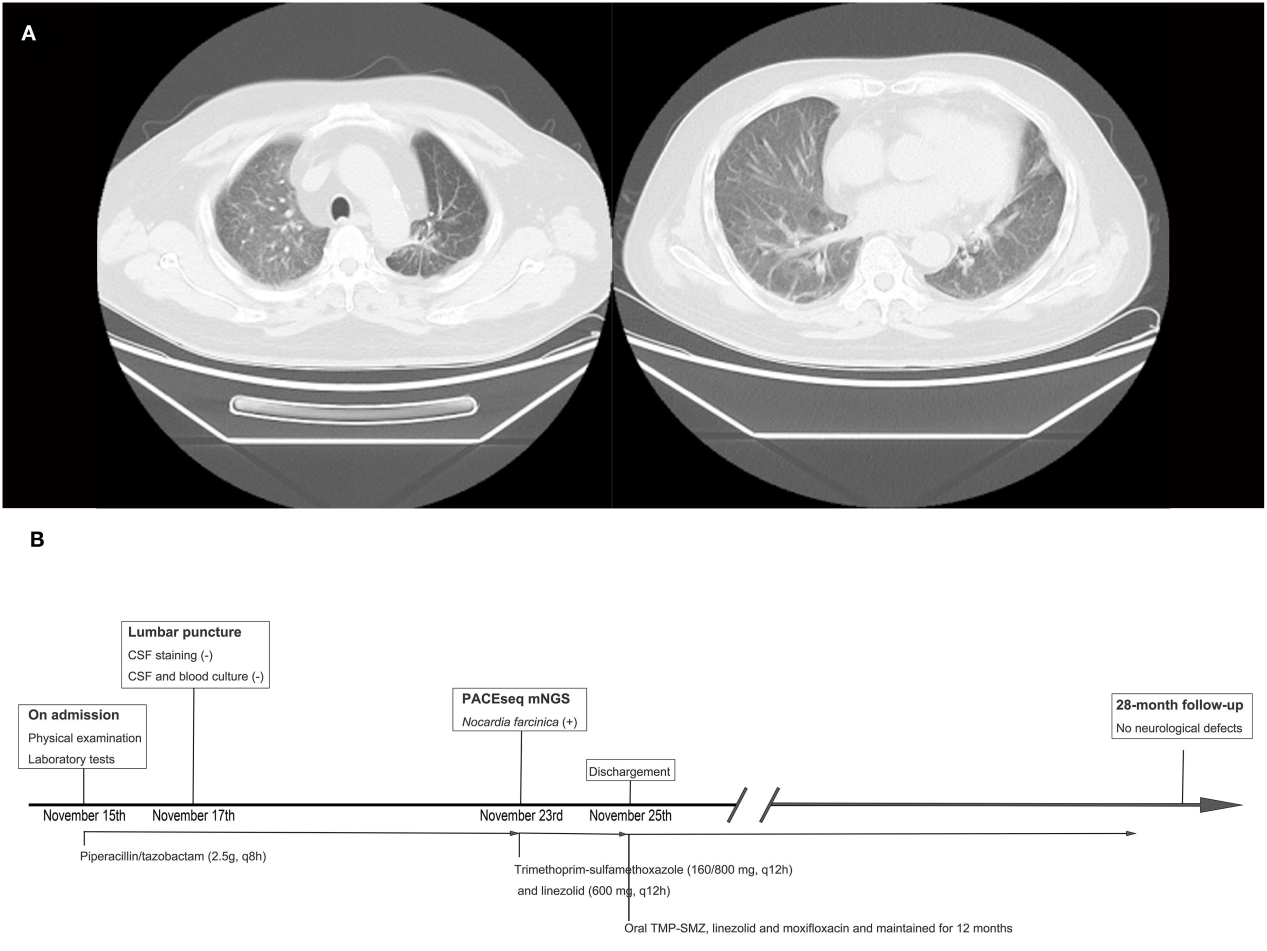
Thoracic computed tomography (CT) scan results and timeline of the treatment. **(A)** Thoracic CT scan showing chronic interstitial inflammation and localized emphysema of the left upper lung. **(B)** Timeline of the treatment of this case.

Laboratory findings included an initial leukocyte count of 9.85 ×10^9^/L (with 88.1% polymorphonuclear cells, 5.7% monocytes, and 6.1% lymphocytes), ESR of 10 mm (normal range 0–20 mm per hour), C-reactive protein of 51 mg/L (normal range <10 mg/L), and albumin level of 41.3 g/L. Brain MRI showed cerebellar vermis and right cerebellar mass with peripheral contrast-enhancement and surrounding edema (Figure 1). Chest CT scan revealed chronic interstitial inflammation and localized emphysema of the left upper lung (Figure 2A). Antibacterial treatment with piperacillin/tazobactam (2.5 g q8h) was empirically started on November 15, 2018.

Lumbar puncture was then performed on November 17, 2018. The pressure was 210 mm H_2_O (normal range 80–180 mmH_2_O). The appearance of the CSF is colorless and clear. CSF analysis demonstrated leukocyte at 7,000 cells/ml, glucose levels at 3.2 mmol/L (normal range 2.8–4.5 mmol/L), and protein levels at 92.4 mg/dl (normal range 8–43 mg/dl). CSF staining including Gram stain, ink stain, and acid-fast stain were all negative. Blood culture and CSF culture were both negative.

**Figure 3:**
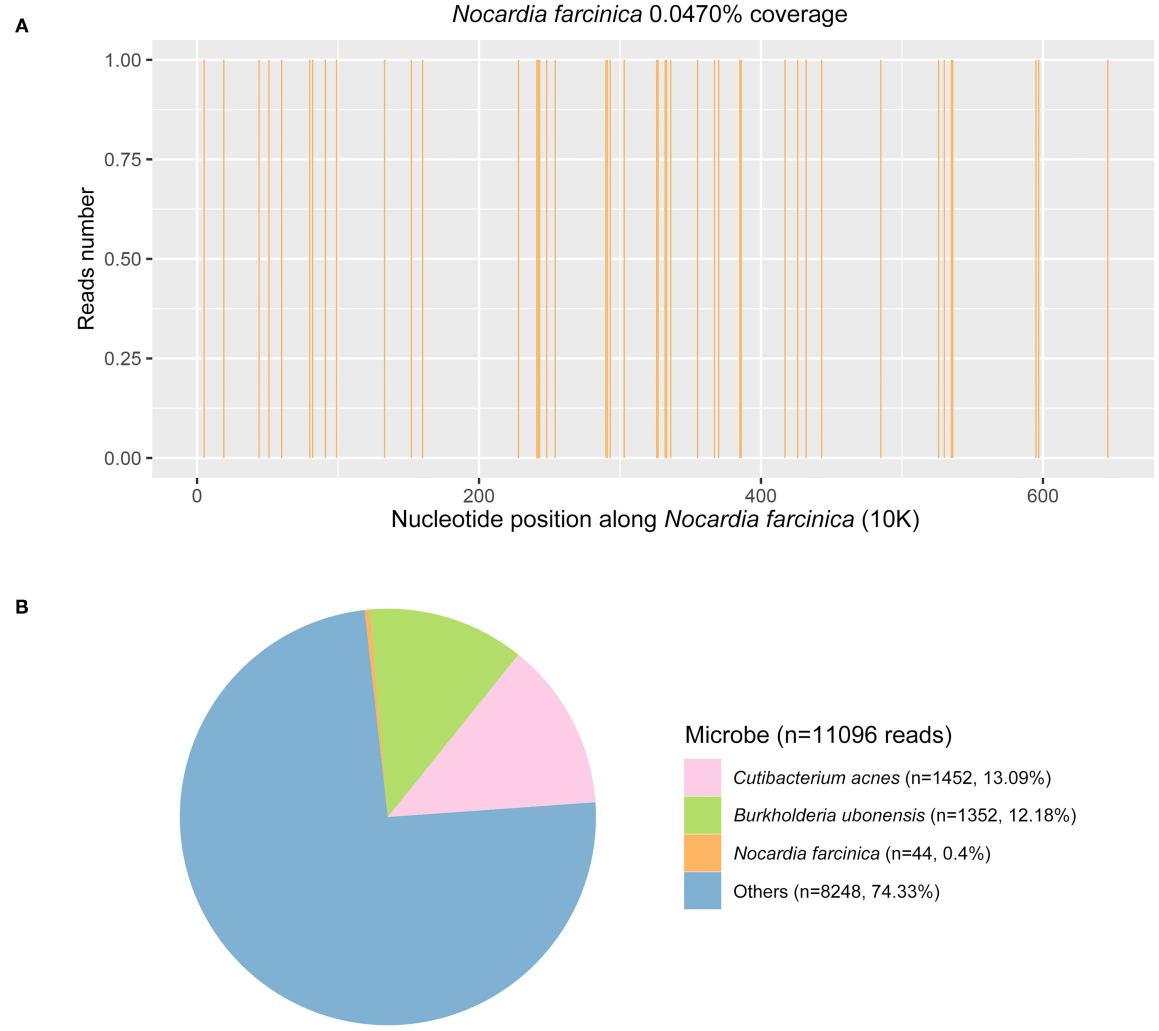
Metagenomic next-generation sequencing (mNGS) results of this case. **(A)** Coverage of *Nocardia farcinica* detected by mNGS was.05%. **(B)** A total of 44 specific reads of *N. farcinica* were detected by mNGS in this case.

PACEseq mNGS test (Hugobiotech, Beijing, China) using CSF sample was performed to identify the pathogens on November 23, 2018. QIAamp DNA Micro Kit (QIAGEN, Hilden, Germany) was used for DNA extraction, and a library of total DNA was built with QIAseq™ Ultralow Input Library Kit for Illumina (QIAGEN, Hilden, Germany). Qubit (Thermo Fisher, MA, USA) and Agilent 2100 Bioanalyzer (Agilent Technologies, Santa Clara, USA) were used to assess the quality of the DNA library. The qualified library was finally sequenced on a Nextseq 550 platform (Illumina, San Diego, USA). After sequencing, adapters and short, low-quality, and low-complexity reads were removed from the raw data. Human DNA was also filtered out by mapping to the human reference database (hg38). The remaining reads were finally aligned to the Microbial Genome Database (ftp://ftp.ncbi.nlm.nih.gov/genomes/). *N. farcinica* (44 specific reads), human cytomegalovirus (2 specific reads), and human herpesvirus 4 (4 specific reads) were detected (Figure 3). The antibacterial regimen was changed to trimethoprim-sulfamethoxazole (TMP-SMZ) (160/800 mg q12h) and linezolid (600 mg q12h). Prednisone and voriconazole were continued to be used in the treatment of pemphigus and fungal pneumonia, respectively. The symptoms of the patient improved significantly, and he was discharged on November 25.

The antibiotic regimen was changed to oral TMP-SMZ, linezolid, and moxifloxacin, and maintained for 12 months. He returned to the hospital for follow-up on March 3, 2019. MRI showed a significant reduction of brain abscess (Figure 1). The patient was followed up for 28 months and was currently asymptomatic without neurological defects. The timeline of the treatment is shown in Figure 2B.

## Discussion and Conclusions

To the best of our knowledge, this is the first case of nocardial brain abscess with pemphigus diagnosed by mNGS without surgical interference. Huang et al. (7) reported a case of brain abscess caused by *Nocardia asiatica* and detected with the combination of Ziehl-Neelsen stain and mNGS. After craniotomy, mNGS identified the pathogen in the purulent fluid from the core of abscess as *N. asiatica*. Another case reported by Zhou et al. (8) showed a idiopathic thrombocytopenic purpura patient with *N. farcinica* brain abscess diagnosed by mNGS using CSF. Unfortunately, the patient was transferred to a local hospital for treatment and lost contact.

The result of mNGS showed *N. farcinica* (44 specific reads), human cytomegalovirus (2 specific reads), and human herpesvirus 4 (4 specific reads). *Cutibacterium acnes* and *Burkholderia ubonensis* were detected as background bacteria. The role of human herpesvirus 4 (Epstein-Barr virus) in central nervous system infections is not fully resolved. EBV associated neurologic diseases include encephalitis, meningitis, cerebellitis, polyradiculomyelitis, transverse myelitis, cranial and peripheral neuropathies, and psychiatric abnormalities. They are usually seen in immunocompromised patients. EBV is commonly found together with other CNS infections including varicella zoster virus, cytomegalovirus, *Cryptococcus*, and *Nocardia*. Immunosuppressive state and other infections in the CNS may trigger EBV reactivation (9). Imaging findings reported on EBV encephalitis are nonspecific. Brain magnetic resonance imaging (MRI) can show normal parenchyma, leptomeningeal enhancement, and multifocal areas of hyperintensity within the subcortical white matter and deep gray nuclei (10). Cytomegalovirus infection of CNS may cause encephalitis, ventriculitis, polyradiculitis, or polyradiculomyelitis (11). Neuroimaging abnormalities include nonspecific white matter changes, periventricular enhancement, and, occasionally, mass lesions with edema and enhancement that mimic tumors (11). In this case, the MRI of the patient showed cerebellar vermis and right cerebellar mass with peripheral contrast enhancement and surrounding edema, and the symptoms of the patient significantly improved after treatment with TMP-SMZ, linezolid, and moxifloxacin, not a combination of antiviral medicines, supporting the diagnosis of brain abscess caused by *Nocardia*.

*Nocardia* is an opportunistic microorganism that exists in nature. It can be inhaled into host. *Nocardia* affects the lungs more frequently than the central nervous system (12). Male sex has been reported to be a risk factor for nocardial infection (13). Brain abscess caused by *N. farcinica* usually occurs in immunocompromised individuals, such as those under long-term use of immunomodulatory therapy, infected with HIV, and those who underwent cancer chemotherapy. Chronic corticosteroid therapy, especially at higher doses and prolonged duration, is associated with increased risk of opportunistic infections such as pneumocystis pneumonia.

The diagnosis of *N. farcinica* brain abscess mainly depended on bacterial culture because of its lack of characteristic imaging and clinical features. Low positive rate of bacterial culture may lead to misdiagnosis and craniotomy. It can be misdiagnosed as ischemic or hemorrhagic stroke, glioma, primary brain tumor, or metastatic malignancy (8, 14).

In this case, the patient was male with a history of pemphigus and had impaired immune function after long-term use of methylprednisolone. The pathogen of CSF was diagnosed to be *N. farcinica* by mNGS. These results suggest that mNGS is a noninvasive and reliable method for pathogen identification. As a result, the patient received timely diagnosis and sensitive antibiotic treatment with good prognosis.

Case reports and small sample cohorts described the clinical features, treatment, and prognosis of nocardial brain abscess (5, 14). Nocardial brain abscess occurs in people aged 50–60 years (15). Our patient is 58 years old. Brain abscess caused by *N. farcinica* tends to have multiple lesions (16, 17). In this case, the right cerebellum and cerebellum vermis were involved simultaneously.

Although limited cases have been reported, in most cases, surgical excision and aspiration are considered necessary for diagnosis and treatment. Surgical aspiration is recommended in lesions larger than 2.5 cm in diameter or if the abscess does not decrease in size within 4 weeks (15). TMP-SMZ is the first line of therapy, and the duration is 6–12 months, or 1 year if CNS is involved. Daily TMP-SMZ prophylaxis may prevent nocardiosis and other opportunistic sensitive infections (17), while TMP-SMZ treatment may not be helpful in the differential diagnosis of nocardia brain abscess. Due to the high morbidity and mortality of nocardial brain abscesses, TMP-SMZ is usually used in combination with another highly bioavailable antibiotic with good penetration to central nervous system (CNS) for the therapy of nocardial brain abscess. Antibiotics with stronger potency, namely, imipenem, amikacin, and linezolid, are recommended for the initial therapy. Recent studies have shown that linezolid can be used in combination with TMP-SMZ for its good CNS penetration (18). In this case, the patient received a combination of TMP-SMZ and linezolid regimen during admission and it was changed to oral TMP-SMZ, linezolid, and moxifloxacin for 1 year.

In conclusion, the possibility of infection with *Nocardia* should be kept in mind in patients who are in immunocompromised condition and exhibiting CNS infection, especially those who lack response to conventional antibiotics. CSF mNGS detection accompanied by conventional blood and CSF culture and fast-acid staining are necessary for early and accurate diagnosis. Empirical therapy including sulfonamides in combination with other high blood-brain barrier penetration antibiotics may prevent high mortality.

## Data Availability Statement

The datasets presented in this study can be found in online repositories. The names of the repository/repositories and accession number(s) can be found below: https://ngdc.cncb.ac.cn/, PRJCA007099.

## Ethics Statement

The studies involving human participants were reviewed and approved by the Ethics Committee of the Affiliated Ganzhou Hospital of Nanchang University. The patients/participants provided their written informed consent to participate in this study.

## Author Contributions

JY and SX participated in the collection of data and drafted the manuscript. JL collected the data for case presentation. XL reviewed the literature and participated in its design. All the authors read and approved the final version of the manuscript.

## Conflict of Interest

HX is employed by Hugobiotech Co., Ltd. The remaining authors declare that the research was conducted in the absence of any commercial or financial relationships that could be construed as a potential conflict of interest.

## Publisher's Note

All claims expressed in this article are solely those of the authors and do not necessarily represent those of their affiliated organizations, or those of the publisher, the editors and the reviewers. Any product that may be evaluated in this article, or claim that may be made by its manufacturer, is not guaranteed or endorsed by the publisher.

## References

[1] Torres OH Domingo P Pericas R Boiron P Montiel JA Vázquez G. Infection caused by Nocardia farcinica: case report and review. Eur J Clin Microbiol Infectious Dis. (2000) 19:205–12. 10.1007/s100960050460 10795594

[2] Corti ME Villafañe-Fioti MF. Nocardiosis: a review. Int J Infectious Dis. (2003) 7:243–50. 10.1016/S1201-9712(03)90102-0 14656414

[3] Mamelak AN Obana WG Flaherty JF Rosenblum ML. Nocardial brain abscess: treatment strategies and factors influencing outcome. Neurosurgery. (1994) 35:622–31. 10.1227/00006123-199410000-00007 7808604

[4] Cassir N Million M Noudel R Drancourt M Brouqui P. Sulfonamide resistance in a disseminated infection caused by Nocardia wallacei: a case report. J Med Case Rep. (2013) 7:103. 10.1186/1752-1947-7-103 23577983 PMC3633055

[5] Corsini Campioli C Castillo Almeida NE O'Horo JC Challener D Go JR DeSimone DC. Clinical presentation, management, and outcomes of patients with brain abscess due to nocardia species. Open Forum Infectious Dis. (2021) 8:ofab067. 10.1093/ofid/ofab067 33855101 PMC8026153

[6] Gu W Deng X Lee M Sucu YD Arevalo S Stryke D. Rapid pathogen detection by metagenomic next-generation sequencing of infected body fluids. Nat Med. (2021) 27:115–24. 10.1038/s41591-020-1105-z 33169017 PMC9020267

[7] Huang T Chen Y Zhang J He R Qu D Ye Q. Rapid and accurate diagnosis of brain abscess caused by Nocardia asiatica with a combination of Ziehl-Neelsen staining and metagenomics next-generation sequencing. Eur J Neurol. (2021) 28:355–7. 10.1111/ene.14533 32920981

[8] Zhou C Wang K Li H Zhang X. Idiopathic thrombocytopenic purpura with brain abscess caused by Nocardia farcinica diagnosed using metagenomics next-generation sequencing of the cerebrospinal fluid: a case report. BMC Infectious Dis. (2021) 21:380. 10.1186/s12879-021-06071-1 33892637 PMC8066483

[9] Martelius T Lappalainen M Palomäki M Anttila VJ. Clinical characteristics of patients with Epstein Barr virus in cerebrospinal fluid. BMC Infectious Dis. (2011) 11:281. 10.1186/1471-2334-11-281 22018204 PMC3213057

[10] Tselis AC. Epstein-Barr virus infections of the nervous system. Handbook Clin Neurol. (2014) 123:285–305. 10.1016/B978-0-444-53488-0.00013-4 25015491

[11] Marra CM. Other central nervous system infections: cytomegalovirus, Mycobacterium tuberculosis, and Treponema pallidum. Handbook Clin Neurol. (2018) 152:151–66. 10.1016/B978-0-444-63849-6.00012-8 29604973

[12] Crozier JA Andhavarapu S Brumble LM Sher T. First report of Nocardia beijingensis infection in an immunocompetent host in the United States. J Clin Microbiol. (2014) 52:2730–2. 10.1128/JCM.00588-14 24829230 PMC4097741

[13] Gorevic PD Katler EI Agus B. Pulmonary nocardiosis. Occurrence in men with systemic lupus erythematosus. Arch Internal Med. (1980) 140:361–3. 10.1001/archinte.1980.00330150075019 7362355

[14] Hofmeister MG Dao A Young AM. Worsening Stroke Symptoms in an 80-Year-Old Man. JAMA Neurol. (2016) 73:1017–8. 10.1001/jamaneurol.2016.0421 27270887

[15] Nicolosi A Hauser WA Musicco M Kurland LT. Incidence and prognosis of brain abscess in a defined population: Olmsted County, Minnesota, 1935-1981. Neuroepidemiology. (1991) 10:122–31. 10.1159/000110257 1922645

[16] Zhu JW Zhou H Jia WQ You J Xu RX. A clinical case report of brain abscess caused by Nocardia brasiliensis in a non-immunocompromised patient and a relevant literature review. BMC Infectious Dis. (2020) 20:328. 10.1186/s12879-020-05052-0 32381049 PMC7206790

[17] Yang M Xu M Wei W Gao H Zhang X Zhao H. Clinical findings of 40 patients with nocardiosis: a retrospective analysis in a tertiary hospital. Exp Therapeut Med. (2014) 8:25–30. 10.3892/etm.2014.1715 24944592 PMC4061227

[18] Rupprecht TA Pfister HW. Clinical experience with linezolid for the treatment of central nervous system infections. Eur J Neurol. (2005) 12:536–42. 10.1111/j.1468-1331.2005.01001.x 15958094

